# A Rare Case of Opsoclonus Myoclonus Ataxia Syndrome Post Viral Illness

**DOI:** 10.7759/cureus.40396

**Published:** 2023-06-14

**Authors:** Nauka S Shah, Jaya Pathak, Purva C Shah, Ummayhany F Bharmal, Maliha I Ansari

**Affiliations:** 1 Department of Medicine, Baroda Medical College, Maharaja Sayajirao (MS) University, Vadodara, IND; 2 Department of Internal Medicine, Baroda Medical College, Maharaja Sayajirao (MS) University, Vadodara, IND; 3 Department of Internal Medicine, Pramukhswami Medical College, Anand, IND

**Keywords:** dancing eyes-dancing feet syndrome, opsoclonus myoclonus, post-viral syndrome, gait ataxia, kinsbourne syndrome, opsoclonus myoclonus ataxia syndrome

## Abstract

Opsoclonus myoclonus ataxia syndrome (OMAS) is a rare inflammatory neurological disorder characterized by ocular, motor, behavioral, language, and sleep disturbances. It usually affects infants and young children but may affect adults. A 28-year-old male was brought to our emergency ward with complaints of involuntary spontaneous eye movements and jerky movements of limbs with imbalance while walking. He had a history of short febrile illness 10 days prior. His magnetic resonance imaging (MRI) of the brain, cerebrospinal fluid (CSF) analysis, and other routine investigations were normal. The patient was treated with injectable methylprednisolone (1 g) given for five days along with other supportive therapy. A significant reduction in the opsoclonus, myoclonus, and ataxia was seen on a six-month follow-up. OMAS should be identified early to avoid the use of inappropriate medications, and immunotherapy must be provided as early as possible in order to prevent irreversible neurological damage.

## Introduction

Opsoclonus myoclonus ataxia syndrome (OMAS), widely known as Kinsbourne syndrome or dancing eyes-dancing feet syndrome, was first described by Orzechowski about a century ago [1]. It is a rare inflammatory neurological disorder characterized by ocular, motor, behavioral, language, and sleep disturbances with a worldwide incidence of one in a million [2]. It has a slight male preponderance and usually affects infants and young children; the peak age of incidence is 18 months, and it is uncommon before six months of age [3]. In adult patients, 50% of OMAS cases are a paraneoplastic presentation of small cell lung cancer, breast cancer, or ovarian cancer [4]. OMAS can also occur as a consequence of infections, drugs and toxins, metabolic disorders, or demyelinating and structural neurological disorders [3].

Most adult cases of OMAS are idiopathic or preceded by a viral (Coxsackie B3 or Saint Louis encephalitis virus) prodrome [4]. Fifty percent of the children affected with OMAS have an underlying tumor, which is neuroblastoma in 50% of cases [5]. Other common malignancies associated with OMAS are cancers of the lung, breast, and gastrointestinal tract; melanoma; and hematological cancers [5]. Compared to OMAS as a part of paraneoplastic syndrome, patients with idiopathic OMAS are younger, more often preceded by prodromal symptoms, and associated with less encephalopathy and have a better outcome and a lower relapse rate [5].

The purpose of this case report is to raise awareness of this rare neurological movement disorder and to help in its early diagnosis and treatment.

## Case presentation

A 28-year-old male was brought to our emergency ward with complaints of involuntary spontaneous eye movements, jerky movements of limbs, and imbalance while walking. These symptoms progressed gradually over the course of 10 days. He was afebrile and did not have headaches, myalgia, or skin rash. He had a history of multiple episodes of nausea and vomiting and a complaint of disturbed sleep for 4-5 days. He had a short febrile illness 10 days prior for which he had taken treatment from a local center. He was relatively healthy 10 days prior. The patient has no history of substance abuse and/or alcohol abuse. Further details of investigation and treatment at the time of presentation were not available.

On general examination, the patient was vitally stable (blood pressure of 110/70 mmHg, pulse of 80/minute, and oxygen saturation of 98% on room air). He was majorly alert with intermittent lethargy and oriented to time, place, and person with a low monotonous speech and reduced spontaneous movements. He had uncontrolled, rapid, and unpredictable eye movement, along with eyelid fluttering, which was suggestive of opsoclonus (Video 1).

**Video 1:** Patient with opsoclonus myoclonus ataxia syndrome (OMAS) at presentation

There was no nuchal rigidity, ophthalmoplegia, or cranial nerve palsy. His Glasgow Coma Scale (GCS) at presentation was 15/15. His pupils were round, regular, and reactive to light. Muscle tone was normal, power was +5 (normal), and superficial and deep reflexes in all limbs were intact. Plantar reflex was flexor on both lower limbs. The patient had generalized involuntary muscle contractions in the body, which aggravated on active movement, suggestive of myoclonus. Intentional tremors, dysarthria, and pendular knee jerk were absent on the examination of cerebellar function. The cardiovascular, respiratory, and gastrointestinal systems were normal on examination. An otolaryngology consult for vestibular neuronitis reported normal eighth nerve function.

Complete blood count (CBC, with peripheral smear), complete metabolic panel (CMP), virology panel, and cerebrospinal fluid (CSF) analysis results were normal. Erythrocyte sedimentation rate (ESR, 28 mm/hour) and C-reactive protein (CRP, 25 mg/L) were mildly elevated (Table 1) [6].

**Table 1:** Laboratory investigations of the patient at presentation g/dL, gram per deciliter; million/mm^3^, million cells per cubic millimeter; %, percent; mg/dL, milligram per deciliter; mmol/L, millimole per liter; mm/hour, millimeter per hour; mg/L, milligram per liter; mOsmol/L, milliosmole per liter; mmH_2_O, millimeter of water; NAD, nicotinamide adenine dinucleotide

Investigation	Results	Reference Range
Blood indices		
Hemoglobin (g/dL)	14.5	13.3-16.2
Red blood cell (RBC) count (million/mm^3^)	5.4	4.3-5.6
Total leukocyte count (×10^3^/mm^3^)	7	3.54-9.06
Neutrophils (%)	68	40-70
Lymphocytes (%)	27	20-50
Monocytes (%)	6	4-8
Eosinophils (%)	2	0-6
Basophils (%)	1	0-2
Platelet counts (×10^3^/mm^3^)	150	165-415
Peripheral smear	NAD	
Liver function tests		
Serum alanine transaminase (ALT) (units/liter)	26	7-41
Serum aspartate transaminase (AST) (units/liter)	32	12-38
Serum creatinine (mg/dL)	0.9	0.6-1.2
Blood urea nitrogen (BUN) (mg/dL)	24	6-24
Serum electrolytes		
Sodium (Na) (mmol/L)	136	136-146
Potassium (K) (mmol/L)	4.1	3.5-5.0
Chloride (Cl) (mmol/L)	105	102-109
Calcium (Ca) (mg/dL)	10.2	8.7-10.2
Magnesium (Mg) (mg/dL)	1.9	1.5-2.3
Erythrocyte sedimentation rate (ESR) (mm/hour)	28	0-15
C-reactive protein (CRP) (mg/L)	25	<10
Infection		
Serum human immunodeficiency virus (HIV) antigen	Negative	Negative
Serum HIV antibody	Negative	Negative
Serum hepatitis B surface antigen (HbSAg)	Negative	Negative
Serum anti-hepatitis C virus (HCV) antibody	Negative	Negative
Venereal disease research laboratory (VDRL) test for syphilis	Negative	Negative
Cerebrospinal fluid (CSF) analysis		
Osmolarity (mOsmol/L)	294	292-297
pH	7.33	7.31-7.34
Glucose (mg/dL)	47	40-70
Total protein (mg/dL)	26	15-50
CSF pressure (­mmH_2_O)	110	50-180
Leukocytes (total) (mononuclear cells per µL)	2	0-5

Magnetic resonance imaging (MRI) of the brain was performed using T1, T2, diffusion-weighted imaging (DWI), gradient axial, T2 sagittal, and fluid-attenuated inversion recovery (FLAIR) coronal sequences. It did not show any significant diagnostic intracranial abnormality. A plain radiograph of the chest, an ultrasonography (USG) of the abdomen and pelvis, and a computed tomography (CT) of the abdomen and pelvis were conducted for the screening of primary tumor and were negative. Further investigations for paraneoplastic panel and virology were not possible due to the financial constraints of the patient.

Based on the clinical history of short febrile illness, examination, investigations, and a neurology consult, the most plausible diagnosis was post-viral OMAS. The patient was treated with methylprednisolone given intravenously (IV) as pulses of 1 g for five days along with other supportive therapies including intravenous (IV) fluids, multivitamins, and calcium.

After five days, the patient’s ocular movements and myoclonic jerks significantly reduced, and he could walk without support (Video 2).

**Video 2:** Improvement in symptoms after treatment OMAS: opsoclonus myoclonus ataxia syndrome

He had persistent horizontal gaze-evoked nystagmus and mild ataxia. Following discharge from our hospital, he was given oral prednisolone (1 mg/kg/day), which was gradually tapered over three months. He was called for follow-up after six months, and there was no residual neurological deficit, nystagmus, or abnormal movements.

## Discussion

The underlying mechanisms responsible for the pathophysiology of OMAS remain poorly elucidated; nevertheless, a potential autoimmune origin has been postulated in such individuals [7]. Autoantibodies are generated against cerebellar neurons and Purkinje cells as a result of molecular mimicry between tumors or infectious agents and cerebral cells. The identification of oligoclonal bands, B-cell activating factor (BAFF), quantitative abnormalities of immunoglobulin G (IgG), and perturbed cytokine profile in the cerebrospinal fluid (CSF) collectively indicate the presence of an autoimmune response [5]. Additionally, literature reports have described mild cellular loss accompanied by inflammatory alterations in the Purkinje cell layer, inferior olives, and brainstem [8].

The typical clinical features of OMAS include opsoclonus (involuntary, chaotic, multi-vector eye movements that are not interrupted by inter-saccadic intervals), myoclonus, ataxia, behavioral problems, and other features of encephalopathy. Acute-onset vertigo, vomiting, and gait instability are the most common presenting symptoms, often leading to the misdiagnosis of peripheral vertigo [3]. Ataxia is actually due to myoclonic jerks (patients may have normal finger-to-nose and heel-to-shin tests); others have prominent cerebellar signs, making it similar to acute cerebellar ataxia [3]. It remains to be seen whether these are different for the subtypes of OMAS.

The diagnosis of OMAS relies on history and clinical examination. Underlying tumors can be detected by contrast tomography (CT) or magnetic resonance imaging (MRI) of the abdomen, chest, neck, and pelvis or positron emission tomography (PET) scan in adults. To detect neurological inflammation or infection, cerebrospinal fluid (CSF) analysis along with antibody detection, lymphocyte subset analysis, and immunophenotyping is performed. A study of inflammatory cytokines and chemokines revealed the presence of increased B-cell attractants (C-X-C motif chemokine ligand 13 {CXCL13}) and B-cell activators (BAFF) in CSF [5]. Since the antigen that triggers OMAS is yet to be identified, serology plays little role in its diagnosis [5].

The treatment goal consists of early and complete neurological remission. After toxic, metabolic, and structural factors are ruled out, neurological remission can be achieved by immunotherapy even in the absence of antibody markers. A three-drug regimen developed by the National Pediatric Myoclonus Center called FLAIR, which consists of high-dose adrenocorticotropic hormone (ACTH), intravenous immunoglobulins (IVIg), and rituximab (monoclonal antibody), shows excellent results for moderate to severe and severe cases [9]. ACTH is replaced with pulse-dosed dexamethasone for mild to moderate cases [9]. Some studies show that prednisone-type steroids are the least effective, while others demonstrate that prednisone and dexamethasone are effective in autoimmune etiology, and the efficacy of treatment increases when given with IVIg [9]. For recurrent and relapsing cases, a biomarker-guided approach of anti-B-cell monoclonal antibody is an effective novel method [5], as individuals who do not respond to primary treatment have higher CSF lymphocytes [10]. Rituximab, an anti-cluster of differentiation 20 (CD20) monoclonal antibody, is gaining popularity in the recent times as it reduces the requirement of steroids and also improves prognosis by reducing the risk of incapacitation [11]. However, it is not free from side effects as it increases infection risk and increases tumor growth [12]. In the case of an underlying tumor, surgical resection followed by OMAS treatment is continued for one to two years [4,5].

The relapse rate with conventional therapy is 50%-75% and much lower with FLAIR [13]. Failure to achieve complete neurological remission and multiple relapses may result in chronic progressive OMAS [2,13]. Neurological complications are more common in the pediatric population [14,15] than in adults, in whom residual dysarthria and gait ataxia take the form of chronic illness [16].

Our patient had all features of OMAS with a history of viral fever with a normal MRI and CSF analysis. Detailed CSF and serum analysis for viral infections and antibodies, as well as the paraneoplastic panel, was not possible due to financial constraints. But the patient responded to immunotherapy with significant improvement [17]. The prognosis of OMAS depends on the cause, being worse with paraneoplastic etiology and favorable in para/post-infectious or idiopathic OMAS, with good response to immunotherapy (mainly IVIg or corticosteroids) [18], suggesting the long-term monitoring of the prognosis for the post-viral OMAS to be unnecessary.

## Conclusions

This was a case of a 28-year-old male with complaints of involuntary spontaneous eye movements and jerky movements of limbs with imbalance while walking and a history of febrile illness. MRI of the brain, CSF analysis, and other routine investigations were inconclusive following which the patient was treated with injectable methylprednisolone along with other ongoing supportive therapies. His symptoms were significantly reduced five days later, and the patient was discharged.

Hence, OMAS should be identified early to avoid inappropriate and unnecessary investigations such as the N-methyl-D-aspartate (NMDA) panel and CSF analysis in some cases and medications and provide immunotherapy as early as possible to the patient. Screening for tumors is important for management strategy, but it is frequently seen to be idiopathic or post-viral. Starting immunotherapy early is prudent to improve prognosis.

## References

[1] Pranzatelli MR (1992). The neurobiology of the opsoclonus-myoclonus syndrome. Clin Neuropharmacol.

[2] (2023). National Organization for Rare Disorders: opsoclonus-myoclonus syndrome. https://rarediseases.org/rare-diseases/opsoclonus-myoclonus-syndrome/.

[3] Caviness JN, Forsyth PA, Layton DD, McPhee TJ (1995). The movement disorder of adult opsoclonus. Mov Disord.

[4] Klaas JP, Ahlskog JE, Pittock SJ (2012). Adult-onset opsoclonus-myoclonus syndrome. Arch Neurol.

[5] Armangué T, Sabater L, Torres-Vega E (2016). Clinical and immunological features of opsoclonus-myoclonus syndrome in the era of neuronal cell surface antibodies. JAMA Neurol.

[6] Kasper D, Fauci A, Hauser S, Longo D, Jameson J, Loscalzo J (2015). Harrison's principles of internal medicine.

[7] Oh SY, Kim JS, Dieterich M (2019). Update on opsoclonus-myoclonus syndrome in adults. J Neurol.

[8] Connolly AM, Pestronk A, Mehta S, Pranzatelli MR 3rd, Noetzel MJ (1997). Serum autoantibodies in childhood opsoclonus-myoclonus syndrome: an analysis of antigenic targets in neural tissues. J Pediatr.

[9] de Alarcon PA, Matthay KK, London WB (2018). Intravenous immunoglobulin with prednisone and risk-adapted chemotherapy for children with opsoclonus myoclonus ataxia syndrome associated with neuroblastoma (ANBL00P3): a randomised, open-label, phase 3 trial. Lancet Child Adolesc Health.

[10] Borker A, Choudhary N (2011). Rituximab. Indian Pediatr.

[11] Pranzatelli MR, Tate ED, Travelstead AL (2009). Insights on chronic-relapsing opsoclonus-myoclonus from a pilot study of mycophenolate mofetil. J Child Neurol.

[12] Chang BH, Koch T, Hopkins K, Malempati S (2006). Neuroblastoma found in a 4-year-old after rituximab therapy for opsoclonus-myoclonus. Pediatr Neurol.

[13] (2019). Genetic and Rare Diseases Information Center: opsoclonus-myoclonus syndrome. https://rarediseases.info.nih.gov/diseases/10009/opsoclonus-myoclonus-syndrome.

[14] Brunklaus A, Pohl K, Zuberi SM, de Sousa C (2011). Outcome and prognostic features in opsoclonus-myoclonus syndrome from infancy to adult life. Pediatrics.

[15] Mitchell WG, Davalos-Gonzalez Y, Brumm VL (2002). Opsoclonus-ataxia caused by childhood neuroblastoma: developmental and neurologic sequelae. Pediatrics.

[16] Honnorat J (2016). New findings in adult opsoclonus-myoclonus syndrome. JAMA Neurol.

[17] Markakis I, Alexiou E, Xifaras M, Gekas G, Rombos A (2008). Opsoclonus-myoclonus-ataxia syndrome with autoantibodies to glutamic acid decarboxylase. Clin Neurol Neurosurg.

[18] Glatz K, Meinck HM, Wildemann B (2003). Parainfectious opsoclonus-myoclonus syndrome: high dose intravenous immunoglobulins are effective. J Neurol Neurosurg Psychiatry.

